# Supplementary material for: Hypoxia Affects HIF-1/LDH-A Signaling Pathway by Methylation Modification and Transcriptional Regulation in Japanese Flounder (Paralichthys olivaceus)
Source: Biology (Basel). 2022 Aug 18;11(8):1233. doi: 10.3390/biology11081233 (PMC9405012; doi:10.3390/biology11081233)
Supplement: Supplementary file 1 [file biology-11-01233-s001.zip › biology-1720151-supplementary.pdf]

Supplementary Materials

Table S1. Primer sequences for methylation-specific PCR (MS-PCR), quantitative real-time PCR (q-PCR), double in situ hybridization (D-ISH) and dual-luciferase reporter assay.

Primer name	Sequence (5′ to 3′)	Product size (bp)	Annealing temperature (℃)	Accession number
HIF-1α (MS-PCR)	F: TAGTAGTATTGTTTTAGAAAGTGAA<br>R: AACAAACCAAAATTAAAACTCTCTA	234	50.7	109628061
LDH-A (MS-PCR)	F: GGGATATAAGGTATGATTGTTTTATT<br>R: TCACACACAACTATTTATATTTCCTA	243	57.0	109638975
HIF-1α (q-PCR)	F: CATGGACACAGGAATTGTACCC<br>R: ATAGCTGATGGTGAGCCTCATG	196	60.0	XM_020084937.1
LDH-A (q-PCR)	F: TGTCAAGTACAGCCCCAACTGC<br>R: TGGAGCTTCTCTCCCATGAGGT	165	60.0	XM_020102195.1
18S (q-PCR)	F: ATTGACGGAAGGGCACCAC<br>R: ATGCACCACCACCCACAGA	134	60.0	EF126037.1
HIF-1α (D-ISH)	F: cgc-atttaggtgacactatagaagcgGATGTAATGCTTCCCTCCTCG<br>R: ccg-taatacgactcactatagggagacaTGCTACTTCCTACTTCGCTGA	535	57.0	XM_020084937.1
LDH-A (D-ISH)	F: cgc-atttaggtgacactatagaagcgGGCAGCAAGAACAAGGTCACA<br>R: ccg-taatacgactcactatagggagacaGCTGGAAGGGTGGATGTGG	555	57.0	XM_020102195.1

pc3.1~HIF-1α	F: cttggtaccgagctcggatccATGGACACAGGAATTGTACCCG<br>R: ccacactggactagtggatccTCAGTTTACGTGGTCCAGTGCG	2388	60.0	XM_020084937.1
pc3.1~HIF-1β	F: cttggtaccgagctcggatccATGTTCTTCCACTCGGACATGTC<br>R: ccacactggactagtggatccTCACTCATTAAACGAAGGGTAGATTG	2259	60.0	XM_020092959.1
pGL~LDH-A	F: ctatcgataggtaccgagctcTAAGAGGCTGACACCCCAAC<br>R: cagtaccggaatgccaagcttTAATTCCCGGATGAGCACGG	805	62.0	109638975
pGL~Lf2	F: ctatcgataggtaccgagctcCTGCTCCACCAGGGACCATT<br>R: cagtaccggaatgccaagcttTAATTCCCGGATGAGCACGG	415	62.0	109638975
pGL~Lf1	F: ctatcgataggtaccgagctcCCCAGCTCATTGCGTATGC<br>R: cagtaccggaatgccaagcttTAATTCCCGGATGAGCACGG	275	62.0	109638975
pGL~Lf0	F: ctatcgataggtaccgagctcCGTTCACGGAGCAGCCTT<br>R: cagtaccggaatgccaagcttTAATTCCCGGATGAGCACGG	246	62.0	109638975
pGL~Lf2m	F: ctatcgataggtaccgagctcCTGCTCCACCAGGGACCATT<br>R: ATGTTGCCGGTAGGCAGGAT<br>F: ATCCTGCCTACCGGCAACAT<br>R: cagtaccggaatgccaagcttTAATTCCCGGATGAGCACGG	407	62.0	109638975
pGL~LDH-Am	F: ctatcgataggtaccgagctcTAAGAGGCTGACACCCCAAC<br>R: ATGTTGCCGGTAGGCAGGAT<br>F: ATCCTGCCTACCGGCAACAT<br>R: cagtaccggaatgccaagcttTAATTCCCGGATGAGCACGG	797	62.0	109638975

A

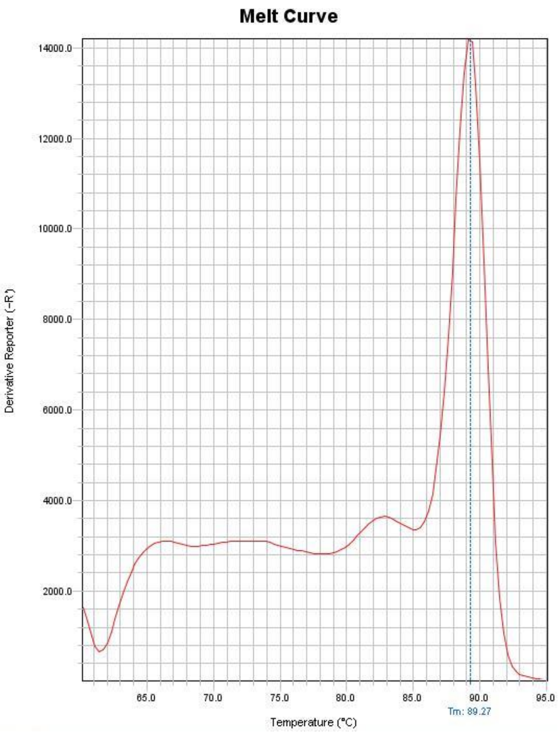
Melt Curve
14000.0
12000.0
10000.0
8000.0
6000.0
4000.0
2000.0
Derivative Reporter (-R')
65.0
70.0
75.0
80.0
85.0
90.0
95.0
Temperature (°C)
Tm: 89.27

B

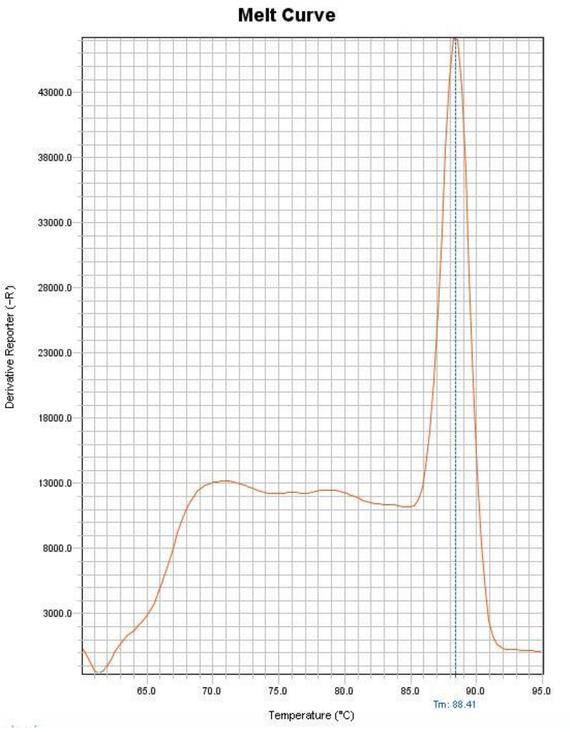
Melt Curve
43000.0
38000.0
33000.0
28000.0
23000.0
18000.0
13000.0
8000.0
3000.0
Derivative Reporter (-R')
65.0
70.0
75.0
80.0
85.0
90.0
95.0
Temperature (°C)
Tm: 88.41

C

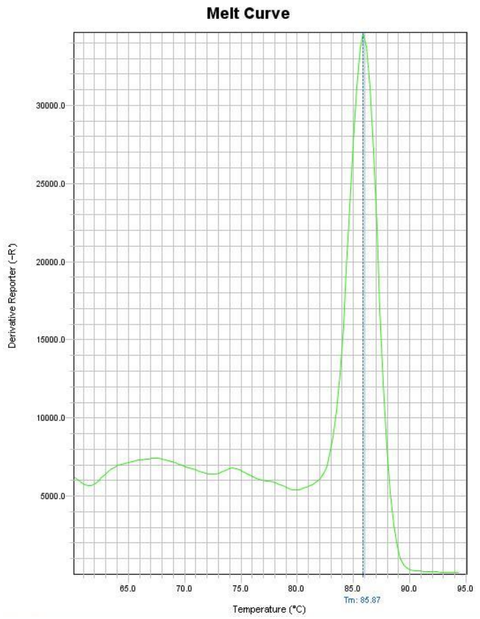

Figure S1. The melt curves of primers for three genes *HIF-1α* (**A**), *LDH-A* (**B**), *18S* (**C**) in q-PCR.

Table S2. The gene sequence analysis results of *HIF-1α* and *LDH-A*.

Gene	Length (bp)	5′ UTR (bp)	Intron (number, bp)	Exon (number, bp)	3′ UTR (bp)	mRNA (bp)	Gene ID
HIF-1α	12737	262	14, 9069	15, 2346	1060	3668	109628061
LDH-A	5999	134	7, 3815	7, 999	1051	2184	109638975

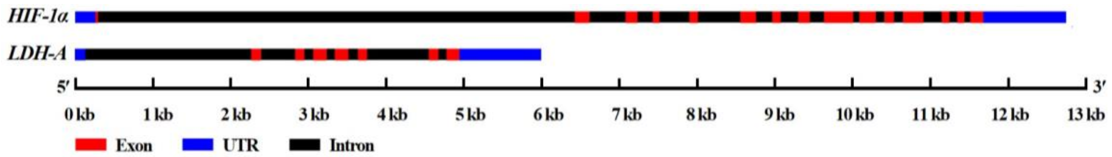

Figure S2. The gene sequence analysis results of *HIF-1α* and *LDH-A*. The red rectangles and black rectangles represent exon and intron sequences, respectively. 5′ UTR and 3′ UTR are expressed by blue rectangles on the left and right, respectively.

Table S3. The protein sequence information of HIF-1α and LDH-A.

Protein	amino acid (aa)	Molecular weight (kDa)	Isoelectric point (pI)	Domain (number)	Transmembrane helices	Accession number
HIF-1α	781	87463.97	4.90	7	0	XP_019940496.1
LDH-A	332	36368.38	7.75	1	0	XP_019957754.1

All "0" in the table indicates none. Values in the column of "Domain" represent the number of domains. Their names and amino acid sites (start - end) were as follows. The 7 domains in HIF-1α protein were Helix loop helix domain (HLH): 22 - 77; PAS domain (PAS): 88 - 154, 230 - 296; Motif C-terminal to PAS motifs (PAC): 302 - 345; Coiled coil region (coiled coil): 355 - 382; Low complexity region (low complexity): 394 - 414, 486-506. The 1 domain in LDH-A protein was low complexity: 25 - 39.

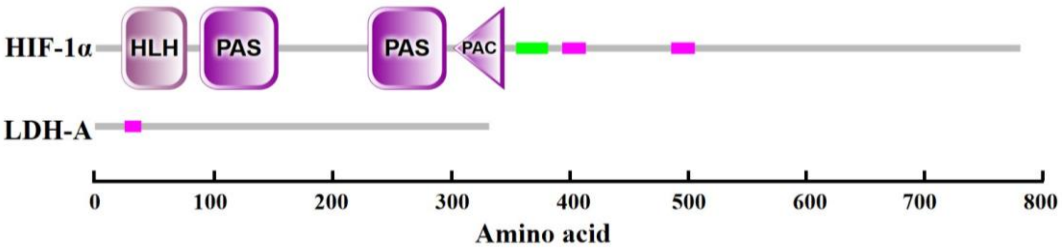

Figure S3. Domain analyses of HIF-1α and LDH-A protein. Domains are same as those in Table S3. The green and magenta boxes represented coiled coil and low complexity, respectively.

A

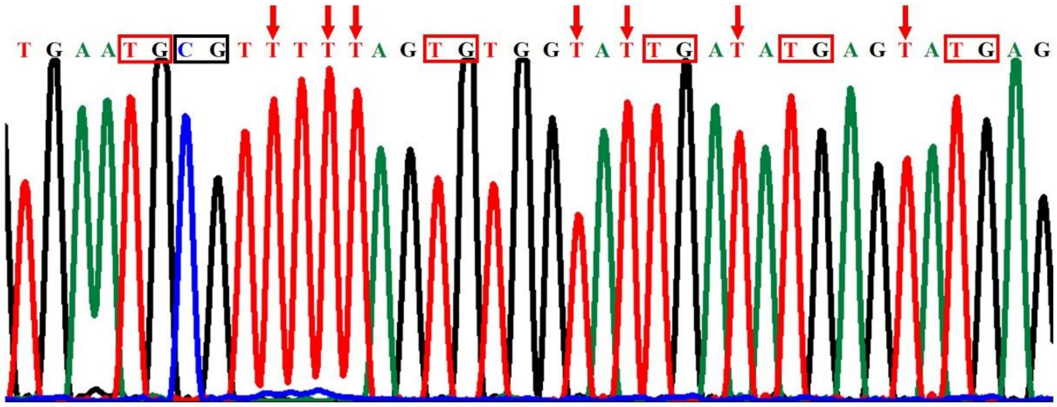

B

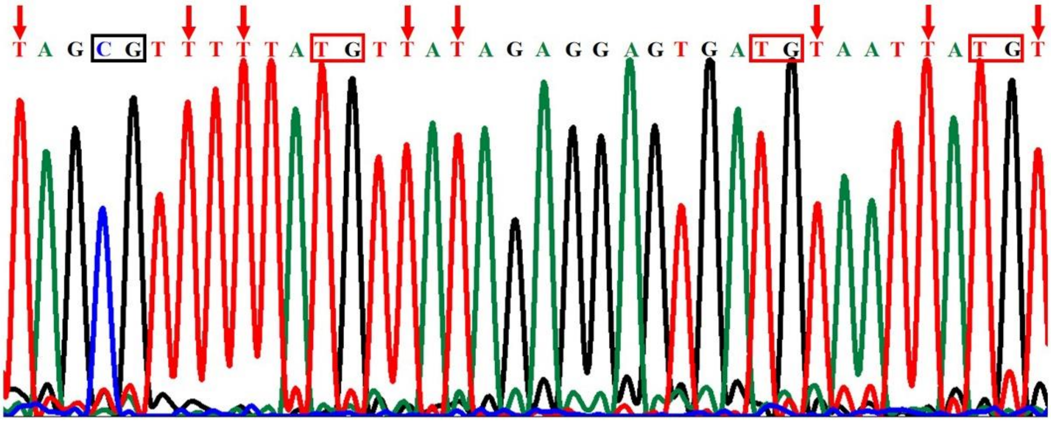

Figure S4. The part DNA sequences of *HIF-1α* gene (**A**) and *LDH-A* gene (**B**) modified by bisulfite. Red arrows denote the bases which are successfully modified from cytosines to thymines by bisulfite. Similarly, the red boxes indicate successfully modified bases which are unmethylated cytosines (changed from cytosines to thymines) in CpG dinucleotides, and the black boxes mean unmethylated cytosines (not changed from cytosines to thymines) in CpG dinucleotides.

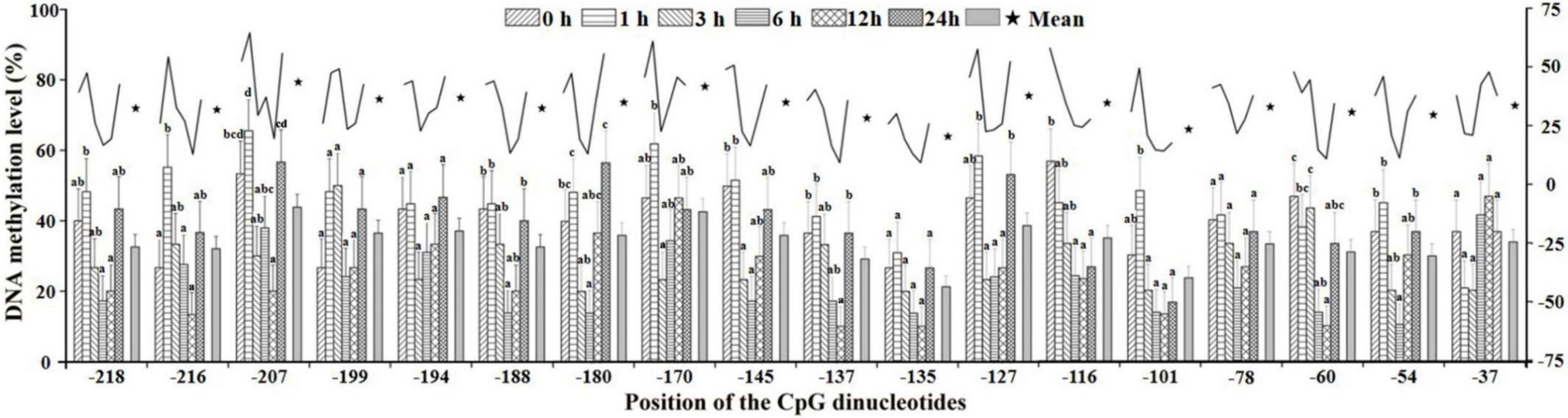

Figure S5. The single CpG dinucleotides methylation level of *HIF-1α* gene in the six treatment groups of Japanese flounder (*Paralichthys olivaceus*). The columns which are following the left ordinate represent the methylation level values of 18 CpG dinucleotides in six hypoxia treatment groups, and the lines following the right ordinate show the methylation level changing trends of them. Furthermore, the five-pointed stars (★) denote the methylation level average values, which follow the right ordinate, of corresponding CpG dinucleotides. In addition, different letters above the columns indicate significant statistical differences ($P < 0.05$).

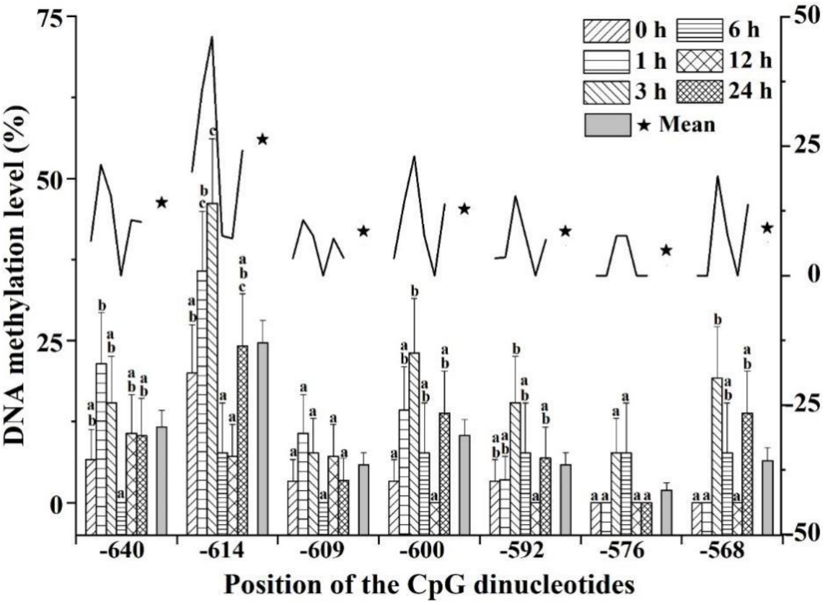

Figure S6. The single CpG dinucleotides methylation level of *LDH-A* gene in the six hypoxia treatment groups of Japanese flounder. The columns which are following the left ordinate represent the methylation level values of 7 CpG dinucleotides in six hypoxia treatment groups, and the lines following the right ordinate show the methylation level changing trends of them. Furthermore, the five-pointed stars (★) denote the methylation level average values, which follow the right ordinate, of corresponding CpG dinucleotides. Different letters above the columns indicate significant statistical differences ($P < 0.05$).

Table S4. Linear regression analysis results of expression and 18 single CpG dinucleotides methylation level in *HIF-1α* gene.

CpG site	R^2	P	Significance level	Function
**-218**	**0.700**	**0.038**	*	**Y = 5.84 - 0.10X**
-216	0.270	0.291	n.s.	---
-207	0.369	0.201	n.s.	---
-199	0.252	0.310	n.s.	---
-194	0.254	0.308	n.s.	---
**-188**	**0.906**	**0.003**	**	**Y = 6.33 - 0.12X**
-180	0.344	0.221	n.s.	---
-170	0.195	0.381	n.s.	---

**-145**	**0.709**	**0.035**	*	**Y = 5.83 - 0.09X**
**-137**	**0.688**	**0.041**	*	**Y = 5.57 - 0.11X**
**-135**	**0.687**	**0.041**	*	**Y = 5.93 - 0.16X**
-127	0.486	0.124	n.s.	---
**-116**	**0.710**	**0.035**	*	**Y = 6.00 - 0.10X**
-101	0.576	0.080	n.s.	---
**-78**	**0.948**	**0.001**	**	**Y = 8.91 - 0.19X**
**-60**	**0.753**	**0.025**	*	**Y = 5.26 - 0.09X**
-54	0.638	0.057	n.s.	---
-37	0.354	0.213	n.s.	---

The word n.s. indicates $P > 0.05$, and the signs *, ** represent $P < 0.05$, $P < 0 .01$ respectively.

Table S5. Linear regression analysis results of expression and 7 single CpG dinucleotides methylation level in *LDH-A* gene.

CpG site	R^2	P	Significance level
-640	0.068	0.618	n.s.
-614	0.012	0.835	n.s.
-609	0.105	0.530	n.s.
-600	0.121	0.498	n.s.
-592	0.188	0.391	n.s.
-576	0.373	0.198	n.s.
-568	0.296	0.265	n.s.

The word n.s. indicates $P > 0.05$.
